# Analysis of the tumor length and other prognosis factors in pT1-2 node-negative esophageal squamous cell carcinoma in a Chinese population

**DOI:** 10.1186/1477-7819-10-273

**Published:** 2012-12-18

**Authors:** Zhengbo Song, Jiwen Wang, Baochai Lin, Yiping Zhang

**Affiliations:** 1 Department of Chemotherapy, Zhejiang Cancer Hospital, 38 Guangji Road, 310022, Hangzhou, People’s Republic of China; 2 Key Laboratory Diagnosis and Treatment Technology on Thoracic Oncology, Zhejiang province, Hangzhou, 310022, China; 3 Department of Thoracic Surgery, Zhejiang Cancer Hospital, Hangzhou, 310022, China

**Keywords:** Esophageal squamous cell carcinoma, TNM classification, Tumor length, Overall survival

## Abstract

**Background:**

Tumor length is an important prognostic factor for many carcinomas, but its role in esophageal cancer remained undetermined. The aim of this study was to investigate the effect of tumor length on survival for patients with confined tumors (grade pT1-2) without lymph-node metastases in esophageal squamous cell carcinoma.

**Methods:**

We enrolled 201 patients with esophageal squamous cell carcinoma (SCC) who had undergone surgical resection and been confirmed as pT1-2N0M0. The relationship of tumor length with overall survival was assessed and compared with other factors detailed in the American Joint Committee on Cancer (AJCC) tumor, node, metastasis (TNM) staging system published in 2009.

**Results:**

The overall survival (OS) rates at 1, 3, and 5 years were 93.0%, 83.7%, and 69.2%, respectively. The tumor length adversely affected OS, with the 5-year rate being 93.5%, 82.0%, 68.6%, 67.9%, 55.3% and 41.1%, respectively for tumor lengths of less than 10 mm, 10 to 20 mm, 20 to 30 mm, 30 to 40 mm, 40 to 50 mm, and greater than 50 mm (*P*< 0.001). Multivariate analyses showed that the pathologic T classification and grade of tumor was significantly associated with OS. Tumor length of 30 mm or more remained an independent prognostic factor (*P* = 0.04), as did the other current TNM factors.

**Conclusion:**

Tumor length appears to affect the OS of patients with early-stage esophageal squamous cell carcinoma. It may provide additional prognostic information for the current TNM staging system.

## Background

Before 1987, the American Joint Committee on Cancer (AJCC) staging system used tumor length to predict patient prognosis [1]; however, the current TNM staging system, first published in 2009, did not take tumor length into account [2]. In the current AJCC staging system for esophageal tumors, stage depends on the depth of the tumor (T classification), lymph-node (LN) involvement (N classification), and distant metastasis (M classification).

Recent publications have suggested that pathologic esophageal tumor length is directly correlated with long-term survival [3-6]; however, most of these data originated in western countries, and the cancer type was predominantly adenocarcinoma.

The aim of this study is to evaluate the value of tumor length and other prognostic factors in predicting the behavior of early-stage esophageal squamous cell carcinoma (SCC) without LN involvement, and the outcome after definitive surgery for such cases, in a Chinese population.

## Methods

We assessed patients who had undergone surgical resection for esophageal cancer between January 2002 and December 2008 in Zhejiang Cancer Hospital. The total number of patients was 1,325, of whom 431 had received a pathological diagnosis of T1-2; 199 of these had LN metastasis, and were excluded. Of the 232 patients without LN metastasis, 13 did not have microscopically tumor-free margin (R0), and a further 18 either had fewer than 12 dissected LNs or had a cancer type other than SCC, and these were also excluded, leaving 201 patients for analysis.

All of the 201 patients with complete tumor resection had pathologically confirmed SCC. The tumor length was measured immediately after resection. Preoperative evaluation and staging investigations included a complete medical history and physical examination, complete blood count and serum biochemistry tests, and scans (for example, computed tomography (CT) of the thorax, ultrasonography of the upper abdomen, magnetic resonance imaging of the brain, total body bone scan, and fluorodeoxyglucosepositron emission tomography).

### Treatment

The extent of the esophageal resection and LN dissection were decided at the time of the operation by the surgeons and took into account the general physical condition of the patient. None of the patients received adjuvant chemotherapy after surgery.

### Follow-up

The surviving patients were followed up every 3 to 6 months for the first 5 years, then annually. Recording of medical history, physical examination, and CT of the chest were performed during the follow-up time. Endoscopic examination and whole-body examination were obtained in cases of clinically indicated recurrence or metastasis. Survival was measured from the date of surgery to the date of death or the final follow-up visit, with. June 2011 being the final censoring date for survival. The median time from surgery to the final censoring date was 52 months (range 30 to 136 months).

### Statistical analysis

The Kaplan–Meier method was used to estimate survival curves. The definition of survival was determined from the date of surgery and the final known follow-up or the date of death. A univariate Cox proportional hazards regression model was used to examine the association between various prognostic predictors and survival. Possible prognostic factors associated with survival probability at a significance level of less than or equal to 0.20 were considered in a multivariable Cox proportional hazards regression analysis. *P*< 0.05 was regarded as significant. All statistical tests were analyzed using SPSS software) version 16.0; SPSS Inc, Chicago, IL, USA).

## Results

### Patient characteristics

Table 1 shows the clinical characteristics of the 201 included patients. The population was predominantly male (86.6%), with a median age of 59 years (range 31–78 years). The most common tumor location was the middle and lower esophagus (94.1%). Of the 201 patients, 115 patients had a tumor length of 30 mm or less and 86 patients had a tumor length greater than 30 mm. The mean and median lengths of the esophageal tumors were 40 and 28 mm, respectively. There were 168 patients who had undergone Ivor-Lewis esophageal resection, and these were categorized as one group for analysis, while patients who had undergone a tri-incisional esophageal resection (n = 25) or any other approach (n = 8) were categorized together as another group. The mean number of LNs dissected was 24.9 (range 13 to 72).

**Table 1:** Demographic characteristics of the study population

**Variable**	
Gender, n (%)	201 (100)
Male	174 (86.6)
Female	27 (13.4)
Age, years	
Range	31 to 78
Median	59
< 65, n (%)	156 (77.6)
≥ 65, n (%)	45 (22.4)
Pathologic T classification, n (%)	
T1	91 (45.3)
T2	110 (54.7)
Grade, n (%)	
Well or moderately differentiated	158 (78.6)
Poorly differentiated or undifferentiated	43 (21.4)
Tumor length	
Mean ± SD	4.0 ± 2.5
≤ 30 mm, n (%)	115 (57.2)
> 30 mm, n (%)	86 (42.8)
Surgical procedure, n (%)	
Ivor-Lewis	168 (83.6)
Tri-incisional	25 (12.4)
Other	8 (4.0)
Tumor location, n (%)	
Upper third	12 (5.9)
Middle third	102 (50.7)
Lower third	87 (43.4)
No. of examined LNs, n (%)	
< 18	29 (14.4)
≥ 18	172 (85.6)

### Analysis by tumor length

Survival time was assessed against increasing length of the tumor in 10-mm increments. As previous reports reported that 30 mm was a well separation [3-6], we used this length to evaluate survival time.

**Figure 1:**
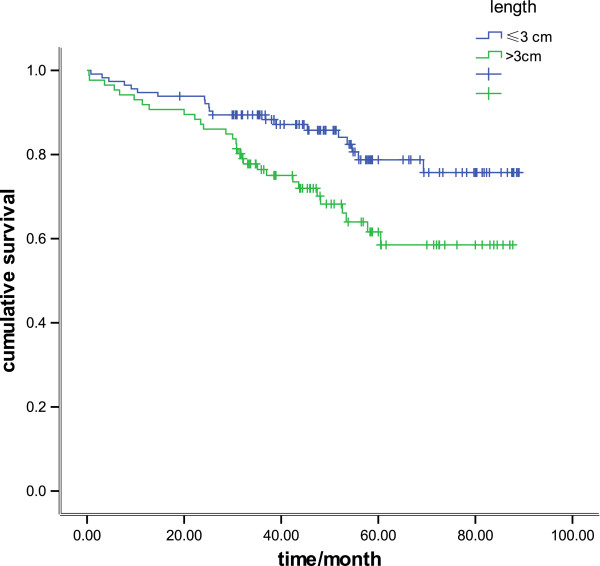
**Kaplan–Meier survival curves in 201 patients with R0-resected esophageal carcinoma, comparing tumor length of 30 mm or less and greater than 30 mm (*P *= 0.01).**

**Figure 2:**
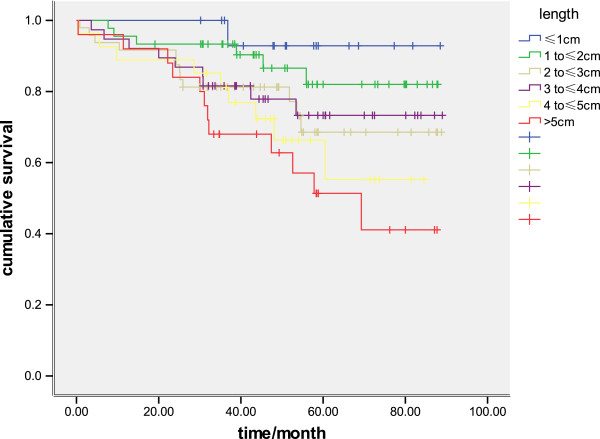
**Kaplan–Meier survival curves in 201 patients with R0-resected early-stage esophageal carcinoma comparing tumor length in 10 mm increments (*P*<0.001).**

**Figure 3:**
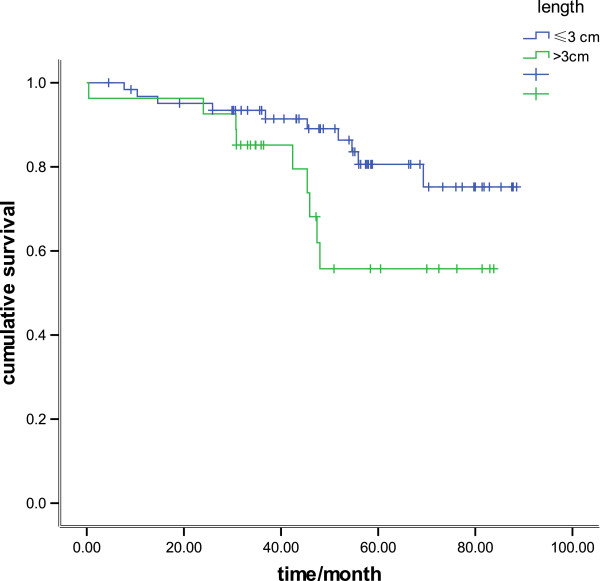
**Kaplan–Meier survival curves in 91 patients assessed as pT1, comparing tumor length of 30 mm or less and greater than 30 mm (*P *= 0.04).**

**Figure 4:**
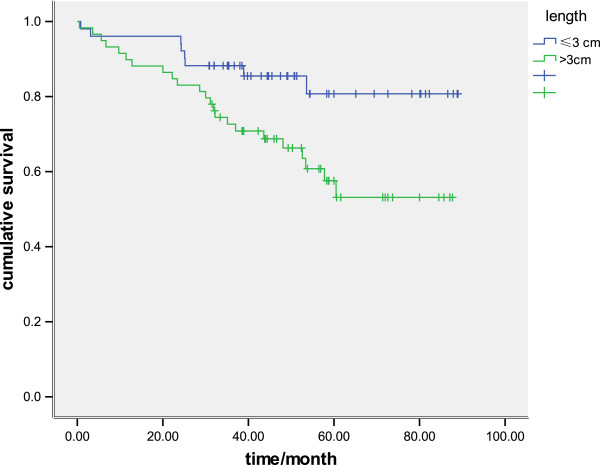
**Kaplan–Meier survival curves in 110 patients assessed as pT2, comparing tumor length of 30 mm or less and greater than 30 mm (P = 0.017).**

There was a significant difference in survival between patients with tumors of 30 mm or less and patients with tumors greater than 30 mm (*P* = 0.01) (Figure 1). When survival was analyzed using size as a continuous variable, there was also a statistical association, with the 5-year survival rate being 93.5%, 82.0%, 68.6%, 67.9%, 55.3%, and 41.1% for tumor lengths of less than 10 mm, 10 to 20 mm, 20 to30 mm, 30 to 4 mm, 40 to 50 mm, and more than 50 mm, respectively (*P*< 0.001) (Figure 2). When survival was analyzed by tumor stage (T1 and T2), the length was also a significant prognostic factor (*P* = 0.04 and *P* = 0.02, respectively: Figure 3, Figure 4). There was a significant difference in 5-year survival in T1a patients when sub-grouped as having tumors of 30 mm or less and tumors greater than 30 mm (91.0% versus 82.5%, *P*< 0.05;),and similar in T1b when sub-grouped as having tumors of 30 mm or less and tumors greater than 30 mm(81.5% versus 72.2%, *P*< 0.04). Similarly, the 5-year survival difference in T2a and T2b patients was also found when they were sub-grouped by tumor size with the cut-off of 30 mm (78.5% versus 71.1%, *P* = 0.04; 65.2% versus 57.2%, *P* = 0.01).

### Factors affecting overall survival assessed by univariate and multivariate analysis

Univariate analyses were performed using the Kaplan-Meier method to assess the predictive capability of each variable assessed (Table 2). As expected, pathologic T classification, tumor grade, and tumor length were predictive of survival. Age, gender, tumor location, surgical procedure, and number of examined LNs were not significantly associated with OS.

**Table 2:** Results of univariate analysis of the prognosis of 201 patients with esophageal carcinoma

**Variable**	**Number**	**Survival,%**		***P *value**
		**3 years**	**5 years**	
Gender				0.23
Male	174	82.4	70.2	
Female	27	92.6	77.8	
Age, years				0.94
≥ 65	45	77.2	74.2	
< 65	156	85	67.6	
Surgical procedure				0.74
Ivor-Lewis	168	83.6	73.5	
Tri-incisional	25	83.8	65.5	
Other	8	87.5	46.7	
Tumor grade				0.05
T1	91	88.8	76.2	
T2	110	79.8	67.4	
Tumor length, mm				0.01
> 30	86	89.5	78.9	
≤ 30	115	76.4	61.6	
Histologic grade				
Well or moderately differentiated	158	86.3	74.3	0.04
Poorly differentiated	43	74.4	60.9	
Tumor location				0.68
Upper third	12	83.3	55.6	
Middle third	102	86	74.3	
Lower third	87	81.4	70.1	
Lymph-node size, mm				0.33
≥ 180	172	84.6	72.9	
< 180	29	79.2	62.3	

Variables used in the final model included age, gender, tumor location, pathologic T stage, surgical procedure, number of examined LNs and tumor length (Table 3). A multivariate Cox regression model was constructed to incorporate age, gender, histologic grade, and the tumor depth, location, and length (30 mm or less versus more than 30 mm). Tumor length (30 mm or less versus more than 30 mm), tumor depth, and histologic grade remained as independent prognostic factors (Table 3), but age, gender, and tumor location did not have a significant influence on survival in multivariate analysis.

**Table 3:** Terms included in the final multivariate model for the prediction of survival

**Variable**	**Hazard ratio**	**95% confidence interval**	**P value**
Gender	0.510	0.182 to 1.433	0.20
Age	0.774	0.389 to 1.540	0.47
Surgical approach	1.076	0.494 to 2.344	0.85
Tumor classification	1.698	1.192 to 2.985	0.04
Tumor length	2.161	1.178 to 3.965	0.04
Histologic grade	2.016	1.081 to 3.763	0.03
Tumor location	0.893	0.543 to 1.468	0.65
Number of examined LNs	0.514	0.228 to 1.159	0.11

LN, lymph node.

## Discussion

To our knowledge, this is one of the largest studies to evaluate the value of tumor length in predicting patient prognosis for resectable early-stage esophageal SCC. We found that pathologic esophageal tumor length was associated with long-term survival in esophageal SCC. Our results suggest that tumor length might be a valuable prognostic factor and might help to identify a high-risk group of patients after surgery for early-stage esophageal SCC.

According to the guidelines of the National Comprehensive Cancer Network, systemic chemotherapy is not recommended for esophageal carcinoma of pT1-2 without LN metastases; observation after complete tumor resection is the recommended approach. However, the 5-year survival rate for some patients, thus it could be useful to assess possible risk factors in addition to tumor depth and grade. In other cancers, such as lung and breast cancers, tumor size is an important prognostic factor for predicting long-term survival, where as only tumor depth and grade are considered prognostic factors in the p-TNM staging for esophageal carcinoma [7].Eloubeidi *et al*. [4] proposed a revised TNM classification for esophageal carcinoma to include tumor length. Other authors have also indicated the importance of tumor length for prognosis; however, most of these data were based on western populations, with the tumor type being predominantly adenocarcinoma [8-10].

In our study, we attempted to eliminate any influence of LN metastases by focusing only on the pathologic esophageal tumor length. In addition, we selected only patients who had undergone esophagectomy with complete tumor R0 resection achieved; any cases withoutR0 resection was excluded. Our study clearly demonstrates that tumor length is an independent predictor of long-term survival, and association of tumor length with survival was consistent regardless of whether length was analyzed as a continuous or categoric variable (Figure 1, Figure 2). In addition, this effect was present even when the other standard staging prognostic factors (pTNM) were taken into account.

The question of how many LNs should be dissected has been a point of debate in previous studies [11-13]. Greenstein *et al*. [14]. and Yang *et al*. [15] recommended 18 as the minimum number of resectable LNs, whereas Peyre *et al*. [16] recommended a minimum of 23 regional LNs, and Schwarz and Smith recommended at least 30 LNs be removed for an adequate lymphadenectomy [17]. However, most previous studies were included patients with locally advanced cancers. The LN dissection number was not clear in early-stage esophageal carcinoma. We did not find any survival difference when using 18 LNs as a cut-off point in our data.

As noted by other authors, Tumor depth also correlated with long-term outcome in patients with different pT disease stages, as reported previously by other authors [9,11]. The overall 5-year survival rates for pT1 and T2 were 76.2% and 67.4%, respectively in this study. Similarly, level of differentiation (well/moderately versus poorly/not) was also a strong prognosis factor in our study (*P* = 0.04).

Our study has several limitations. It was a retrospective study with all the limitations that accompany such a study. In addition, because the study used data from a single institution but with different pathologists and different surgeons, there may have been a lack of uniformity in measurement methods. The LN dissection number was also not consistent, and we also excluded patients who had a dissected LN number of less than 12, which may have influenced our analysis. Thus, carefully designed prospective studies are needed to confirm the role of tumor length in guiding esophageal SCC treatment.

## Conclusion

In summary, our study suggests that esophageal tumor length in early-stage esophageal SCC without LN involvement could be a useful prognostic factor to help identify high-risk patients with esophageal cancer before surgical resection. We found that esophageal tumor length was a significant independent predictor of long-term survival. We consider that it should be incorporated in the current esophageal cancer staging system to better predict the long-term survival of patients with esophageal cancer.

## Competing interests

The authors declare that they have no competing interests.

## Authors’ contributions

YZ and ZS cooperated in the conception and design of the study, and in the collection of the data;JW and BL validated all pathology reports, and assisted in data analysis and interpretation of data; andZS drafted the manuscript. All authors approved the final manuscript.
